# Healthy days at home and prognosis of older adults with cancer and non-cancer serious life-limiting illnesses

**DOI:** 10.1186/s12877-025-06160-9

**Published:** 2025-07-03

**Authors:** Oluwaseun J. Adeyemi, Nina Siman, Allison M. Cuthel, Keith S. Goldfeld, Corita R. Grudzen

**Affiliations:** 1 Department of Emergency Medicine, New York University Grossman School of Medicine, New York, 10016 USA; 2 Department of Population Health, New York University Grossman School of Medicine, 10016 New York, USA; 3 Division of Supportive and Acute Care Services, Memorial Sloan Kettering Cancer Center, 10065 New York, USA

**Keywords:** Prognosis, Healthy days at home, Serious life-limiting illness, Cancer, Gagne score

## Abstract

**Background:**

Healthy Days at Home (HDaH) is a patient-centered outcome measure quantifying the number of days individuals spend at home without hospitalizations or emergency department (ED) visits, while maintaining functional independence. This study examines the association between HDaH and prognosis among US older adults with serious life-limiting illnesses (commonly heart failure, chronic obstructive pulmonary disease, advanced cancer, and end-stage kidney disease) and explores how this relationship differs by cancer status.

**Methods:**

For this prospective cohort design study, we pooled Medicare Claims data of older adults (aged 66 or greater) with serious life-limiting illnesses who visited one of 30 EDs participating in the Primary Palliative Care for Emergency Medicine study between 2015 and 2019. The main exposure was prognosis, measured using the Gagne index, a short-term predictor of mortality. We controlled for age, sex, race/ethnicity, and used cancer diagnosis as a secondary predictor and stratification variable. The outcome, HDaH, was defined as 180 days minus the days a patient spent in healthcare institutions, including hospitals, skilled nursing facilities, and hospice care. We used generalized linear mixed-effects models with a log (180) offset to estimate the adjusted rate ratios (aRR) and 95% confidence intervals.

**Results:**

The cohort included 122,579 seriously ill older adults,11% (*n* = 13,452) of whom had cancer. The median (IQR) HDaH was 115 (26–174) days. Each unit increase in Gagne index score was associated with a 6.0% decrease in the rate of HDaH (aRR: 0.94; 95% CI: 0.94 to 0.94), a pattern observed in both cancer and non-cancer groups. Cancer diagnosis was associated with 7.0% increase in HDaH (aRR: 1.07; 95% CI: 1.07 to 1.07).

**Conclusion:**

While poor prognosis is associated with fewer healthy days at home, cancer diagnosis is associated with more healthy days at home. Our findings highlight the need for tailored care models to reduce hospitalizations and increase HDaH for patients with serious life-limiting illnesses other than cancer.

## Introduction

Approximately 75% of U.S. older adults with serious life limiting illnesses visit the emergency department (ED) in the last six months of life, with three quarters of these individuals being admitted to the hospital [1]. Common serious, life-limiting illnesses commonly include heart failure, chronic obstructive pulmonary disease (COPD), advanced cancer, or end-stage kidney disease [2, 3]. ED visits and subsequent hospital admissions often represent pivotal moments in illness trajectories accelerating functional decline, worsening frailty, and increasing the likelihood of future ED revisits [4–7]. Although many of these patients ultimately die in the hospital, [1] studies indicate that some older adults with serious illnesses prefer less aggressive treatment and prioritize spending time at home with loved ones while receiving quality care [8–10].

In this context, Healthy Days at Home (HDaH) and prognosis have emerged as important concepts for assessing and guiding care among older adults with serious life-limiting illnesses. HDaH is a patient-centered outcome measure that captures the number of days individuals spend at home without hospitalizations or ED visits [11, 12]. This metric shifts the focus from traditional health utilization measures to a more holistic assessment of well-being, particularly for older adults with serious life-limiting illnesses [12]. Evidence suggests that interventions such as early discharge planning, palliative care consultations, and timely outpatient follow-up can reduce hospital stays, reduce readmission rates, and increase HDaH [13–16].

Prognosis, on the other hand, defined as the expected course and outcome of a medical condition, [17] helps clinicians understand the likely progression of an illness and the potential impacts of interventions. The Gagne index is a prognostic tool that stratifies individuals by short-term mortality risk, defined as mortality within 180 days of follow-up after hospital discharge or ambulatory care visit [18]. Patients with a Gagne index greater than six, [18] indicating high short-term mortality risk, often experience recurrent complications requiring ED visits and hospitalizations, [19] which reduce their ability to remain at home. Conversely, poor prognosis in an older adult often prompts serious illness conversations to identify care strategies that align with patient preferences [20, 21]. These preferences may include avoiding aggressive treatments and prioritizing comfort measures, [22, 23] potentially increasing HDaH. The relationship between prognosis and HDaH, therefore, remains unclear.

Differences in healthcare utilization related to prognosis also exist between cancer and non-cancer populations with serious life-limiting illnesses [24–26]. Patients with advanced cancer often engage in more structured prognostic discussions and receive palliative care, which can improve care transitions and increase HDaH [27]. In contrast, older adults with non-cancer illnesses, such as advanced heart failure or COPD, frequently experience less clear prognostic trajectories, resulting in fragmented care and fewer opportunities for proactive serious illness conversations [24, 28, 29]. The possibility exists that the structured prognostic and palliative care discussions, more commonly received by patients with advanced cancer diagnosis, [30, 31] may be a contributing factor in differential healthcare utilization practices among patients with cancer and non-cancer serious life-limiting illnesses. Yet, objectively assessing how HDaH vary among patients with cancer and non-cancer serious life-limiting illnesses serves as a critical first step in understanding how practices such as earlier integration of palliative services, routine prognostic conversations, and enhanced outpatient follow-up might be adapted to improve outcomes for patients with cancer and non-cancer serious life-limiting illnesses [32, 33].

Because HDaH is a patient-centered measure, understanding the factors that help older adults with serious life-limiting illnesses spend more healthy days at home may improve the quality of care they receive. Specifically identifying cancer as a factor associated with increased HDaH would justify future studies aimed at isolating the specific care processes that contribute to these outcomes and determining whether diagnosis-specific interventions are necessary to optimize HDaH across diverse patient populations. Furthermore, by examining how prognosis and cancer diagnosis intersect, providers can identify factors that increase HDaH while aligning care strategies with patients’ preferences. This study aims to explore the relationship between HDaH and prognosis in older adults with serious life-limiting illnesses, while controlling for demographic characteristics and stratifying by cancer status.

## Methods

### Study design and population

In this retrospective cohort analysis, we identified older adults who visited at one of 29 emergency departments (EDs) participating in the Primary Palliative Care for Emergency Medicine (PRIM-ER) study. PRIM-ER is a pragmatic, cluster-randomized, stepped-wedge clinical trial designed to assess the impact of primary palliative care implementation in EDs on healthcare utilization among patients with serious, life-limiting conditions. The rationale for using PRIM-ER patient population in this study was to define a clinically relevant cohort of older adults with serious life-limiting illnesses who sought emergency care, while minimizing the variability across different EDs across the US. The EDs enrolled in the PRIM-ER study were selected based on their diversity, leadership willingness, and receptivity to randomization for palliative care intervention [34] Detailed information on the study protocol has been previously published, [35–38] and the study is registered on ClinicalTrials.gov (NCT03424109, Registration Date: 30th January, 2018) [39].

### Data source

Data for this study were extracted from Medicare claims using the Centers for Medicare & Medicaid Services Chronic Conditions Data Warehouse. The Medicare claims database, the largest U.S. healthcare administrative dataset, includes health information on individuals aged 65 and older, as well as for those under 65 with disabilities. For this analysis, we used the Master Beneficiary Summary File A/B/C/D and the inpatient, outpatient, hospice, and carrier claims files. These files were merged using unique beneficiary identifiers to ensure accurate linkage.

### Inclusion and exclusion criteria

We pooled Medicare claims data between January 1, 2014 and June 30, 2019. During this period, there were a total of 147,507 unique Medicare enrollees aged 66 years and older who had an initial ED visit—the first eligible visit for a patient at one of the 30 ED sites. We computed the Gagne score, a prognosis of six-month mortality (180 days) (see Gagne computation below) [18]. Gagne score higher than six correlates with high risk of short-term mortality [18, 19]. Hence, our final analytic sample, therefore, consisted of older adults with high risk of short-term mortality (*n* = 122,579).

### Outcome measure

The primary outcome variable was HDaH. HDaH was defined as the number of days a patient spent outside healthcare institutions, including hospitals, skilled nursing facilities, and inpatient hospice care, over a specified period. HDaH was calculated as 180 days from index hospital admission minus the number of days spent in inpatient hospital stays (including inpatient observation and psychiatry), skilled nursing facilities, ED visits (including outpatient observation), rehabilitation, long term hospital settings, days with home health visits, and mortality day [11]. HDaH was measured as a continuous variable.

### Predictor variables

For this study, the demographic characteristics included age, sex, and race/ethnicity. For our final model, we measured age as a continuous variable, sex as a binary variable (male or female), and race/ethnicity as a five-level categorical variable: non-Hispanic White (hereafter referred to as White), non-Hispanic Black (hereafter referred to as Black), Hispanic, Asian, and other races. Prognosis was defined using the Gagne score, which is derived from the Romano-Charlson Index and the Elixhauser system to predict one-year mortality in older adults. The score was calculated based on the presence of 20 diagnoses, identified using ICD-9 (from January 2014 to September 2015) and ICD-10 (from October 2015 to December 2018) codes from inpatient and outpatient claims. The Gagne score was treated as a continuous variable, with a range from − 3 to 25. For this study, we selected individuals with a Gagne score of 7 or higher as being at higher risk for adverse health outcomes. Cancer diagnosis was included as a secondary predictor variable and a stratification variable. We identified those with a cancer diagnosis using ICD-9 and ICD-10 codes for advanced cancer. We determined a patient’s disease status (Cancer and the 20 diagnoses used for Gagne score computation) based entirely on the 1 year prior to the index visit. No diagnoses were determined following the index admission.

### Human subject concern

Our study used Centers for Medicare and Medicaid Services’ Claims data of older adults with serious life-limiting illnesses. We obtained waiver of consent to use the data given the study’s geographic breadth and volume of eligible patients. The study was performed according to the guidelines in the Helsinki Declaration. This study was approved by the institutional local ethics committee - the NYU Grossman School of Medicine Institutional Review Board (Study ID i18-00607).

### Statistical analyses

We first examined the distribution of the study population by demographic characteristics, prognosis and cancer diagnosis. Next, we computed the summary statistics of HDaH and examined how these days spent at home differ by demographic characteristics, prognosis, and cancer diagnosis. We then used a generalized linear mixed-effects model with a log (180) offset to estimate the association between prognosis and HDaH. We also conducted separate analysis for individuals with and without a cancer diagnosis. We reported the adjusted rate ratios (aRR) and 95% confidence intervals (95% CI) for the association between the predictor variables and HDaH. All analyses were conducted using SAS Version 7.1 and R/Databricks Version 4.1.1, accessed through the Chronic Conditions Warehouse Virtual Research Data Center.

## Results

Our study population included 122,579 seriously ill older adults, 11% (*n* = 13,452) of whom had a cancer diagnosis (Table 1). The mean (SD) age across the patient population was 78.5 (8.4) years, with nearly equal male to female distribution (49.9% vs. 50.1%). The majority of the patient population was White (76.9%), followed by Black (15.9%). The mean (SD) Gagne score for the patient population was 8.7 (2.0).

**Table 1:** Descriptive statistics of the demographic and health characteristics of the study population (*N* = 122,579)

Variables	All Patients (*N* = 122,579)	Cancer Diagnosis (*n* = 13,452; 11.0%)	Non-Cancer Diagnosis (*n* = 109,127; 89.0%)	
Age (Mean, SD)	78.5 (8.4)	77.2 (7.7)	78.7 (8.5)	
Gender (N, %)				
Female	61,401 (50.1)	3,494 (26.0)	57,907 (53.1)	
Male	61,178 (49.9)	9,958 (74.0)	51,220 (46.9)	
Race/Ethnicity (N, %)				
White	94,257 (76.9)	10,488 (78.0)	83,769 (76.8)	
Black	19,546 (15.9)	2,011 (14.9)	17,535 (16.1)	
Hispanic	1,872 (1.5)	177 (1.3)	1,695 (1.6)	
Asian	3,055 (2.5)	287 (2.1)	2,768 (2.5)	
Other Races^a^	3,849 (3.1)	489 (3.6)	3,360 (3.1)	
Prognosis (Mean, SD)				
Gagne Score	8.7 (2.0)	9.3 (2.4)	8.6 (1.9)	

*SD* Standard Deviation

^a^Other Races includes North American Native, Other, and Unknown

When comparing the cancer and non-cancer groups, the mean (SD) age of the cancer population was younger (77.2 [7.7] years) compared to the non-cancer population (78.7 (8.5) years). The cancer group had a higher proportion of males (74.0%) compared to the non-cancer group (46.9%). Additionally, 78.0% of patients in the cancer group and 76.8% in the non-cancer group were White. The mean (SD) Gagne score for cancer patients was slightly higher (9.3 [2.4]) than for non-cancer patients (8.6 [1.9]).

The median (IQR) number of HDaH for the overall population was 114 (27–174) days (Table 2). Among those aged 65–74 years, the median (IQR) HDaH was 124 (34–176) days, which decreased to 115 (27–175) days for those aged 75–84 years, and 95 (18–159) days for ages 85 and older. Males had a slightly higher median (IQR) HDaH of 116 (26–175) days compared to females with 112 (28–173) days. Black patients had the lowest median (IQR) HDaH of 110 (27–173) days. Patients with a Gagne score of 9 or less had a higher median (IQR) HDaH of 122 (37–176) days compared to those with a Gagne score greater than 9 (76 days [11–158]).

**Table 2:** Distribution of health days at home across by demographic and health characteristics of the study population

Variables	HDaH Median (IQR) (in Days)		
	All Population 114 (27–174)	Cancer Diagnosis 120 (27–177)	Non-Cancer Diagnosis 113 (27, 174)
Age Category			
65–74	124 (34–176)	130 (32–178)	124 (34–176)
75–84	115 (27–175)	122 (28–177)	114 (27–175)
85+	95 (18–159)	97 (19–170)	95 (18–158)
Sex			
Female	112 (28–173)	109 (23–176)	112 (29–173)
Male	116 (26–175)	124 (30–177)	114 (25–174)
Race/Ethnicity			
White	114 (26–174)	121 (28–177)	113 (26–174)
Black	110 (27–173)	115 (23–176)	109 (28–172)
Hispanic	123 (41–175)	132 (33–178)	122 (41–175)
Asian	116 (28–175)	106 (28–175)	117 (28–175)
Other Races^a^	130 (36–177)	137 (28–179)	129 (38–177)
Prognosis Score			
Gagne 9 or less	122 (37–176)	139 (44–179)	121 (36–176)
Gagne 9+	76 (11–158)	83 (13–167)	75 (10–156)

*HDaH *Healthy Days at Home ^a^Other Races includes North American Native, Other, and Unknown

Comparing cancer and non-cancer groups, patients with cancer had slightly higher median HDaH (120 days vs. 113 days). Patients with cancer diagnosis aged 65–74 had an HDaH median (IQR) of 130 (32–178) days compared to 124 (34–176) days for those with non-cancer diagnosis. Males with a cancer diagnosis had a higher median (IQR) HDaH of 124 (30–177) days compared to 114 (25–174) days among males with non-cancer diagnosis. Blacks with cancer diagnosis had a higher median (IQR) HDaH of 115 (23–176) days compared to 109 (28–172) days among Blacks without cancer diagnosis. Patients with cancer having worse prognosis (Gagne score 9 or higher) had a higher median (IQR) HDaH of 83 (13–167) days compared to 75 (10–156) days among those without cancer diagnosis but with worse prognosis.

Table 3 summarizes the adjusted regression results for factors predicting the number of healthy days at home. A unit increase in age was associated with a 9% decrease in the rate of healthy days at home (Adjusted Rate Ratio (aRR): 0.91; 95% CI: 0.91 to 0.91), a pattern of association that persisted in both cancer and non-cancer groups. Males with cancer diagnosis had an 8% higher rate of healthy days at home compared to females (aRR: 1.08; 95% CI: 1.07 to 1.08). Blacks had a 2% reduction in the rate of healthy days at home compared to Whites (RR: 0.98; 95% CI: 0.98 to 0.98), with this pattern persisting in the cancer and non-cancer groups. Hispanics had a 4% (aRR: 1.04; 95% CI: 1.03 to 1.06) higher rate of HDaH in the cancer group and a 6% (aRR: 1.06; 95% CI: 1.06 to 1.07) higher rate in the non-cancer group. A unit increase in the Gagne score was associated with a 6.0% decrease in the rate of HDaH (RR: 0.94; 95% CI: 0.94 to 0.94), a pattern that persisted in both cancer and non-cancer groups. Patients with a cancer diagnosis had 7% increased rate of HDaH compared to those without a cancer diagnosis (aRR: 1.07; 95% CI: 1.07 to 1.07).

**Table 3:** Association patient characteristics and HDaH across the entire population and by cancer and non-cancer diagnoses

Variables	All Patients (*N* = 122,579)	Cancer Diagnosis (*n* = 13,452; 11.0%)	Non-Cancer Diagnosis (*n* = 109,127; 89.0%)
	aRR [95% CI]	aRR [95% CI]	aRR [95% CI]
Age (continuous)	0.91 [0.91 to 0.91]	0.92 [0.91 to 0.92]	0.91 [0.91 to 0.91]
Sex			
Female	Ref	Ref	Ref
Male	1.01 [1.00 to 1.01]	1.08 [1.07 to 1.08]	1.00 [1.00 to 1.00]
Race/Ethnicity			
White	Ref	Ref	Ref
Black	0.98 [0.98 to 0.98]	0.97 [0.97 to 0.98]	0.98 [0.98 to 0.98]
Hispanic	1.06 [1.06 to 1.07]	1.04 [1.03 to 1.06]	1.06 [1.06 to 1.07]
Asian	1.01 [1.01 to 1.02]	0.96 [0.95 to 0.98]	1.02 [1.01 to 1.02]
Other Races^a^	1.04 [1.04 to 1.05]	1.01 [1.00 to 1.02]	1.05 [1.04 to 1.05]
Prognosis (continuous)			
Gagne	0.94 [0.94 to 0.94]	0.94 [0.94 to 0.94]	0.94 [0.94 to 0.94]
Diagnosis			
Cancer Diagnosis	1.07 [1.07 to 1.07]		
Non-Cancer Diagnosis	Ref		

*HDaH *Healthy Days at Home, *aRR *Adjusted Rate Ratio ^a^Other Races includes North American Native, Other, and Unknown

## Discussion

Our study provides valuable insights into the relationships between prognosis, demographic factors, and cancer diagnosis in predicting HDaH among older adults with serious life-limiting illnesses. We found that a worse prognosis, as indicated by a higher Gagne score, is associated with reduced HDaH among older adults with serious life-limiting illnesses, a pattern observed in both individuals with and without cancer. Additional predictors of reduced HDaH include older age and being Black, while having a cancer diagnosis and being Hispanic are associated with increased HDaH. Although these patterns are consistent across cancer and non-cancer groups, a notable exception is observed in the Asian population – those with cancer diagnosis have reduced rates of HDaH, while those without cancer diagnosis have higher rates of HDaH, compared to Whites.

As the prognosis of seriously ill older adults worsen, the number of days spent at home decreases. This observed relationship between worsen prognosis and reduced HDaH likely reflects the difference in health status across individuals with different prognoses. A poorer prognosis is often associated with more progressive decline in health status, frequent complications, and reduced functional status, [40] which can lead to more ED revisits, hospital admissions, and greater reliance on acute care services [41]. Patients who are experiencing more acute exacerbations of a disease may be unable to stay at home, even if they desire to do so [42]. Additionally, those with more severe illness may require closer monitoring and more intensive medical management to address emerging complications, often leading to increased hospital visits or prolonged stays, further reducing their number of healthy days they can spend at home.

We found that seriously ill older adults with cancer have more HDaH compared to those with non-cancer diagnoses. This difference may be attributed to the structured and comprehensive care pathways available to cancer patients, such as outpatient oncology services, home-based palliative care, and symptom management plans designed to minimize hospitalizations [43–45]. These tailored interventions support better control of symptoms and complications, enabling cancer patients to spend more time at home. In contrast, individuals with non-cancer diagnoses like advanced heart failure, end-stage kidney disease, or advanced COPD often face unpredictable disease trajectories characterized by acute exacerbations. These sudden declines typically lead to ED visits or hospital readmissions, limiting their ability to remain at home [46–48]. Furthermore, non-cancer conditions may have fewer targeted outpatient and palliative care resources compared to cancer, further restricting opportunities for maintaining HDaH [49]. This disparity highlights the need for more integrated care models for patients with non-cancer serious illnesses to enhance their ability to stay at home.

This study has several limitations. First, HDaH is based on administrative data, which may not fully capture the complexity of patients’ health statuses or their home care environments. Our study population was older adults with serious life-limiting illnesses who presented in the ED and thus excludes those that do not have any recorded ED visits. Additionally, we lack data on socioeconomic status, family support, and patient-reported health outcomes, all of which could confound the association between prognosis and HDaH by either increasing or reducing the observed effect sizes. Our study was limited to older adults who visited one of the 29 participating EDs, which may affect the generalizability of the study. Since our older adult cohort was selected from EDs whose leadership were willing to participate in a palliative care intervention, there may be an increased tendency for these older adults to have longer healthy days at home. However, a recent randomized study found no evidence that palliative care affects healthcare utilization [50]. We identified the cohort with cancer diagnoses using ICD codes, which, although highly specific, have lower sensitivity for advanced cancer, and their sensitivity and specificity can vary across different cancer types, potentially introducing misclassification bias [51, 52]. Lastly, while the HDaH definition assumes that days excluding those spent in hospitals, skilled nursing facilities, EDs, rehabilitation, long-term hospitals, and with home health suggest healthy days at home, the HDaH computation does not account for delays in healthcare access, functional status, quality of life, or individual goals of care. Despite these limitations, the study benefits from its large, diverse sample of older adults with serious life-limiting illnesses and the use of Medicare claims data, the largest US administrative database. While the goal for end-of-life care should center on providing care that aligns with patients’ wishes, our study provides foundational information that healthcare providers can use to understand possible preferences for end-of-life care based on prognosis, diagnosis and demographic characteristics. Future studies can expand upon this work by linking administrative data with electronic health records, providing an opportunity to account for additional sociodemographic variables, and measures of healthcare access, frailty, and quality of life.

## Conclusion

Among US older adults with serious life-limiting illnesses, worse prognosis is associated with fewer HDaH. Increasing age is associated with fewer HDaH, with substantial variability by race/ethnicity. In contrast, cancer is associated with more HDaH. While factors such as age, sex, and race are non-modifiable, more can be done to improve care for older adults with serious non-cancer-based life-limiting illnesses, such as advanced heart failure, COPD, and end stage kidney disease. Our findings highlight the need for tailored care models, including enhanced outpatient services and home-based care to reduce hospitalizations and increase HDaH for patients with serious non-cancer illnesses. Prioritizing patient preferences in care strategies can further promote independence and improve the quality of life for all seriously ill older adults.

## Data Availability

Data for this analysis is not publicly available because it is stored within the Centers for Medicare and Medicaid Services’ Virtual Research Data Center and only accessed by certified users who hold a current virtual seat.

## Abbreviations

ED: Emergency Department
COPD: Chronic Obstructive Pulmonary Disease
HDaH: Healthy Days at Home
PRIM-ER: Primary Palliative Care for Emergency Medicine
